# The Novel Organic Arsenical PZ2 Exhibits Potent Anti-T-ALL Activity via ROS-Dependent Apoptosis and *GABARAPL1*-Induced Autophagic Cell Death

**DOI:** 10.3390/cancers18193150

**Published:** 2026-09-29

**Authors:** Xian Zhang, Zhuping Chen, Yujiao Liu, Dongdong Zhang

**Affiliations:** 1 Department of Cardiology, Qianjiang Central Hospital, Qianjiang 433100, China; 15171842705@163.com; 2 Department of Hematology, Postgraduate Union Training Base of Xiangyang No.1 People’s Hospital, School of Medicine, Wuhan University of Science and Technology, Xiangyang 441000, China; 18727808907@163.com; 3 State Key Laboratory of Separation Membranes and Membrane Processes, School of Chemistry, Tiangong University, Tianjin 300387, China; 4 Department of Oncology, Xiangyang Central Hospital, Hubei University of Arts and Science, Xiangyang 441000, China

**Keywords:** acute T-lymphoblastic leukemia, organic arsenic compound PZ2, *GABARAPL1*, autophagy, ROS, apoptosis

## Abstract

**Objective:** T-cell acute lymphoblastic leukemia (T-ALL) is a highly aggressive hematological malignancy with poor overall prognosis. This study systematically evaluates the anti-leukemic therapeutic potential and underlying redox-driven mechanisms of PZ2, a novel organic arsenic compound, in T-ALL. **Methods:** The cytotoxicity of PZ2 against T-ALL cell lines and normal cells was assessed to determine its therapeutic window. RNA-seq was utilized to map transcriptomic alterations. Intracellular redox homeostasis, mitochondrial membrane potential (MMP), cell cycle, and apoptosis were evaluated using flow cytometry and fluorescence microscopy. Ultrastructural changes and key protein expressions were analyzed via TEM, RT-qPCR, and immunoblotting. In vivo efficacy was validated using a T-ALL cell-line-derived xenograft (CDX) mouse model. **Results:** PZ2 exhibited superior cytotoxicity and an improved safety profile compared to inorganic arsenic trioxide (ATO). Transcriptomic data indicated strong enrichment in apoptosis and autophagy–lysosome pathways. PZ2 induced profound mitochondrial oxidative stress, leading to ROS accumulation, MMP loss, and subsequent activation of caspase-dependent apoptosis—effects that were completely rescued by ROS scavengers. Furthermore, PZ2 triggered G2/M phase arrest and activated autophagic flux (upregulated *GABARAPL1* and LC3-II, degraded p62). Notably, pharmacological inhibition of autophagy rescued PZ2-induced apoptosis, revealing a pro-death autophagic mechanism. In vivo, PZ2 significantly suppressed tumor growth and prolonged survival without overt systemic toxicity. **Conclusions:** PZ2 exhibits potent anti-T-ALL activity that may involve a dual mechanism: ROS-dependent mitochondrial apoptosis and pro-death autophagy potentially associated with *GABARAPL1*. This novel organic arsenical may represent a promising and potentially safer alternative for leukemia therapy, and these findings suggest a potential interplay between oxidative stress and autophagic cell death.

## Simple Summary

This study systematically evaluated the anti-leukemic activity and mechanistic basis of PZ2, a novel organic arsenical, in highly aggressive T-cell acute lymphoblastic leukemia (T-ALL). Our results demonstrated that PZ2 exerted potent cytotoxicity against T-ALL cells and exhibited a superior safety profile compared with inorganic arsenic trioxide. Mechanistically, PZ2 triggered mitochondrial oxidative stress, leading to reactive oxygen species (ROS) accumulation, loss of mitochondrial membrane potential, and subsequent activation of caspase-dependent apoptosis. Concurrently, PZ2 upregulated GABARAPL1 and LC3-II, decreased p62 levels, and activated autophagic flux; pharmacological blockade of autophagy partially reversed PZ2-induced cell death, supporting a pro-death role for autophagy in this context. In vivo, PZ2 significantly suppressed tumor growth and extended survival in mouse xenograft models, with no overt systemic toxicity observed. In conclusion, these findings suggest that PZ2 may act through a dual mechanism—ROS-driven mitochondrial apoptosis and *GABARAPL1*-associated pro-death autophagy—indicating that this compound may serve as a promising therapeutic candidate for T-ALL with potentially improved safety.

## 1. Introduction

T-cell acute lymphoblastic leukemia (T-ALL) is a highly aggressive hematologic malignancy characterized by the abnormal proliferation and widespread infiltration of immature T-lymphoblasts originating in the thymus, which subsequently suppress normal hematopoiesis [1]. While intensified, multi-agent chemotherapy regimens have significantly improved the 5-year overall survival rate to approximately 90% in pediatric T-ALL, outcomes for adult patients and relapsed/refractory cases remain dismal, with long-term survival plunging below 30% [2]. Although allogeneic hematopoietic stem cell transplantation (allo-HSCT) serves as the primary curative modality for high-risk T-ALL, nearly half of the patients succumb to relapse within one year post-transplantation, rendering the overall efficacy unsatisfactory [3,4]. Therefore, identifying novel therapeutic vulnerabilities and developing safer, highly effective targeted agents are urgent clinical imperatives for T-ALL.

Modulating intracellular redox homeostasis has emerged as a promising strategy for overcoming chemoresistance in cancer therapy. Reactive oxygen species (ROS) act as a double-edged sword: physiological low levels function as second messengers driving cellular proliferation and survival, whereas excessive ROS accumulation beyond a critical lethal threshold induces irreversible macromolecular damage, culminating in apoptosis or ferroptosis [5]. In T-ALL, the regulation of the ROS–apoptosis axis is particularly critical. Hyperactivation of oncogenic signaling, such as the NOTCH1 pathway, typically suppresses ROS production to maintain a low-oxidative state, thereby fueling leukemia-initiating cell activity and driving disease progression [6]. Consequently, modern therapeutic strategies aim to deliberately amplify oxidative stress to breach this ROS tolerance threshold, selectively eradicating T-ALL cells [7].

Parallel to apoptosis, autophagy represents another highly conserved mechanism governing cellular fate under stress [8]. While basal autophagy promotes tumor survival by clearing damaged organelles and limiting oxidative damage early in tumorigenesis [9,10], hyperactivated or sustained autophagy can trigger autophagic cell death (pro-death autophagy)—an alternative mechanism to eliminate apoptosis-resistant cancer cells [11,12]. Consequently, anti-cancer strategies are shifting from merely “inhibiting autophagy” to “precisely hyperactivating” it to kill tumors. Central to this process is *GABARAPL1* (GABA Type A Receptor-Associated Protein Like 1), a core mammalian homolog of yeast ATG8 essential for autophagosome formation [13,14]. Although elevated *GABARAPL1* expression correlates with enhanced autophagic cell death and favorable prognoses in solid tumors and acute promyelocytic leukemia (APL) [15], its precise role in T-ALL remains elusive. Recent evidence in acute myeloid leukemia (AML) highlights *GABARAPL1* as a critical epigenetic target for autophagic regulation [16], suggesting its potential as a therapeutic vulnerability in T-ALL, particularly when operating synergistically with apoptosis [17].

Arsenic trioxide (ATO) stands as a paradigm of successful translational medicine, achieving curative outcomes in APL. Beyond degrading the PML-RARα fusion protein, ATO exerts broad-spectrum anti-leukemic effects by robustly inducing ROS, activating apoptosis, and modulating autophagic flux [18,19,20,21,22]. However, its broader clinical application is severely constrained by a narrow therapeutic window, insufficient target specificity, and significant systemic toxicity. To address these clinical limitations, we synthesized PZ2, a novel organic arsenical compound designed with improved chemical stability and a superior safety profile. In this study, we systematically evaluated the therapeutic potential of PZ2 against T-ALL both in vitro and in vivo.

## 2. Materials and Methods

### 2.1. Reagents

The novel organoarsenical compound PZ2 was successfully synthesized via a sequential three-step procedure involving the preparation of intermediate 4-(1,3,2-dithioarsinan-2-yl)aniline and piperoyl chloride, followed by a selective coupling reaction, yielding a final product with a purity of 99.279%. Arsenic trioxide (ATO; As_2_O_3_, MW: 197.84, #A1010) was purchased from Sigma-Aldrich (St. Louis, MO, USA). N-acetylcysteine (NAC; #S0077) and penicillin–streptomycin solution (#C0222) were obtained from Beyotime Biotechnology (Shanghai, China). Dimethyl sulfoxide (DMSO; #D8371-50) was purchased from Solarbio (Beijing, China). 3-methyladenine (3-MA; #M129496) was acquired from Aladdin (Shanghai, China). Fetal bovine serum (FBS; #10099-141C), RPMI-1640 medium (#21870076), trypsin (#25200056), and PBS (#10010023) were sourced from Gibco (Thermo Fisher Scientific, Waltham, MA, USA).

### 2.2. Antibodies

Primary antibodies used in this study included: GABARAPL1 (#A23781) and CTSB (#A19005) from Abcam (Cambridge, MA, USA); p62 (#18420-1-AP), LC3 (#14600-1-AP), KIM-1 (#30948-1-AP), NGAL (#26991-1-AP), GAPDH (#60004-1-Ig), and β-Tubulin (#10094-1-AP) from Proteintech (Wuhan, China); PARP (#9542S), Caspase-3 (#9662), BCL-2 (#4223), BAD (#9292), and BAX (#2772) from Cell Signaling Technology (Danvers, MA, USA). V450-conjugated CD3 antibody (#378512) was obtained from BioLegend (San Diego, CA, USA).

### 2.3. Cell Culture

Human T-ALL cell lines Jurkat (CL-0129), Molt-4 (CL-0160) and CCRF-CEM (CL-0058) were obtained from Procell (Wuhan, China). Mouse embryonic fibroblasts (3T3) were provided by Servicebio (Wuhan, China). Human renal proximal tubule epithelial cells (HK2) were obtained from Fuhang Biotechnology (Shanghai, China). All cells were cultured in RPMI 1640 medium supplemented with 10% FBS and 1% penicillin–streptomycin in a humidified incubator at 37 °C with 5% CO_2_.

### 2.4. Cell Viability Assay

Cell proliferation was evaluated using the Cell Counting Kit-8 (CCK-8; Bioss, Beijing, China). Briefly, T-ALL cells (2 × 10^4^ cells/well) were seeded in 96-well plates and treated with varying concentrations of PZ2. After 24 or 48 h of incubation, 10 μL of CCK-8 solution was added to each well. Following an additional 2–4 h of incubation, absorbance was measured at 450 nm using a microplate reader. Cell viability (%) was calculated as [(ODtreatment − ODblank)/(ODcontrol − ODblank)] × 100%.

### 2.5. Apoptosis Assay

Cellular apoptosis was determined using the Annexin V-APC/PI Apoptosis Detection Kit (Beyotime, Shanghai, China). Cells were seeded in 6-well plates (2 × 10^5^ cells/well) and treated with 0.6 μM PZ2 for 24 h. For rescue experiments, cells were pretreated with NAC (5 mM) or MitoQ (50 nM) for 2 h, or 3-MA (5 mM) for 1 h, followed by PZ2 treatment. After treatment, cells were harvested, washed twice with ice-cold PBS, and stained with APC and PI in 1× binding buffer according to the manufacturer’s protocol. Apoptosis rates were analyzed via flow cytometry (FlowJo software, v11).

### 2.6. Mitochondrial Membrane Potential (MMP) Assay

MMP was assessed using the JC-1 Mitochondrial Membrane Potential Assay Kit (Elabscience, Wuhan, China). After treatment with 0.6 μM PZ2 for 24 h, cells were harvested and incubated with JC-1 working solution at 37 °C for 30 min in the dark. For rescue experiments, cells were pretreated with NAC (5 mM) or MitoQ (50 nM) for 2 h before PZ2 treatment. Stained cells were washed and resuspended in PBS for immediate detection of the red/green fluorescence ratio by flow cytometry.

### 2.7. Mitochondrial ROS Measurement

Mitochondrial ROS levels were determined using an ROS assay kit (Prometheus Biotechnology, Wuhan, China). Cells were incubated with 10 μM H2DCFH-DA probe in serum-free medium for 30 min at 37 °C in the dark. Post-incubation, cells were washed and analyzed for fluorescence intensity by flow cytometry.

### 2.8. Cell Cycle Analysis

Cell cycle distribution was analyzed using the BeyoClick™ EdU-647 Cell Proliferation Kit (Beyotime, Shanghai, China). Briefly, cells treated with PZ2 were incubated with 1× EdU working solution for 2 h, fixed with fixative solution, and labeled with Click reaction solution. Nuclei were counterstained with DAPI, and the cell cycle distribution was determined by flow cytometry.

### 2.9. Quantitative Real-Time PCR (RT-qPCR)

Total RNA was extracted using the RNA extraction kit (Vazyme, Nanjing, China). cDNA synthesis was performed using the All-in-one RT SuperMix (Biopmk, Wuhan, China). qPCR was conducted using SYBR Green qPCR Mix (Biopmk) on a real-time PCR system. Relative gene expression was calculated using the 2^−ΔΔCt^ method, with *GAPDH* serving as the internal control. Primer sequences are detailed as follows: *GABARAPL1*: F: GGACCACCCCTTCGAGTATC, R: CCTCTTATCCAGATCAGGGACC; *CTSB*: F: AGAGTTATGTTTACCGAGGACCT, R: GATGCAGATCCGGTCAGAGA; GAPDH: F: CAAGGTCATCCATGACAACTTTG, R: GTCCACCACCCTGTGCTGTAG.

### 2.10. mRNA Sequencing and Functional Enrichment Analysis

Molt-4 cells were treated with 0.6 μM PZ2 or DMSO for 24 h, with three independent biological replicates. Library preparation (including mRNA purification, fragmentation, cDNA synthesis, and adapter ligation) and sequencing were then performed. Differentially expressed genes (DEGs) were identified using DESeq2 with thresholds of |log_2_FC| ≥ 1 and FDR < 0.05. Principal component analysis (PCA) was used to evaluate overall differences among samples (Supplementary Figure S1). KEGG pathway enrichment analysis and gene set enrichment analysis (GSEA) were performed (Supplementary Figure S2).

### 2.11. Western Blot

Total proteins were extracted using RIPA lysis buffer supplemented with PMSF (Beyotime). Protein concentrations were measured by BCA assay. Equal amounts of protein (15 μg) were separated via 8–12% SDS-PAGE and transferred onto PVDF membranes. Membranes were blocked, incubated with primary antibodies (1:5000) overnight at 4 °C, and then with HRP-conjugated secondary antibodies. Bands were visualized using an ECL detection system (Meilun Biotech, Dalian, China).

### 2.12. Transmission Electron Microscopy (TEM)

After PZ2 treatment for 24 h, cells were collected by centrifugation, washed once with PBS, and fixed with pre-cooled (4 °C) 2.5% glutaraldehyde. Cells were resuspended in the fixative and incubated at room temperature for 30 min in the dark. After three washes with PBS to remove glutaraldehyde, samples were fixed in 1% osmium tetroxide at 4 °C for 1–2 h in the dark. The cells were then dehydrated in acetone, embedded in epoxy resin, sectioned with an ultramicrotome, stained with 2% uranyl acetate and lead citrate, and observed under a transmission electron microscope.

### 2.13. Animal Experiments

All animal experiments were approved by the Animal Care and Use Committee of Hubei University of Medicine (approved number 2022-010). Female NOD-SCID mice (4–5 weeks old) were obtained from Charles River Laboratories (stock no. 406) and maintained under specific pathogen-free (SPF) conditions. After successful engraftment was verified by morphological evaluation of peripheral blood and bone marrow smears, the mice were randomly divided into two groups, with 12 mice in each group. Body weight was measured every 3 days. The treatment group received intraperitoneal injections of PZ2 (3.5 mg/kg/day) for 12 consecutive days, while the control group received an equal volume of corn oil. On day 22, the mice were sacrificed, and blood and bone marrow were collected for flow cytometry analysis. The following CD45 gating strategy was used for flow cytometry: first, a gate was set based on forward scatter (FSC) and side scatter (SSC) parameters to exclude cell debris and dead cells and to define the single viable cell population; subsequently, cells were gated on V450-CD45 fluorescence to define the CD45-positive cell population, which was used to assess Molt-4 cell engraftment in the blood and bone marrow.

### 2.14. Wright–Giemsa Staining for Morphological Analysis

Bone marrow cells were isolated by flushing the femoral cavities with PBS. Peripheral blood samples were collected via tail vein puncture. Smears of both bone marrow cells and peripheral blood were prepared on glass slides, air-dried, and stained with Wright–Giemsa solution (Solarbio, Beijing, China) according to the manufacturer’s instructions. This staining protocol was employed to evaluate cellular morphology and estimate the proportion of leukemic blasts. The slides were examined under an oil-immersion light microscope equipped with a digital camera system to capture representative images of blast cell infiltration.

### 2.15. Immunohistochemistry (IHC)

Mouse spleen tissues were harvested, fixed in 4% paraformaldehyde (PFA) for 24 h, embedded in paraffin, and sliced into 3–5 μm sections. Following deparaffinization in xylene and rehydration through a graded ethanol series, the sections underwent heat-mediated antigen retrieval using a citrate buffer (pH 6.0) in a microwave oven. Endogenous peroxidase activity was quenched by incubation with 3% hydrogen peroxide for 10 min. After blocking with 5% bovine serum albumin (BSA) at room temperature for 1 h to prevent non-specific binding, sections were incubated overnight at 4 °C with primary antibodies against GABARAPL1, CTSB, and LC3B. Following three washes with PBS, the sections were incubated with horseradish peroxidase (HRP)-conjugated secondary antibodies for 30 min at 37 °C. Immunoreactivity was visualized using 3,3′-diaminobenzidine (DAB) chromogen, and sections were counterstained with hematoxylin, dehydrated, and mounted with neutral balsam. Images were captured using a bright-field microscope.

### 2.16. H&E Staining

For histopathological evaluation, spleen tissues were fixed in 4% PFA for 24 h. The fixed tissues were dehydrated through an ascending ethanol series (70%, 80%, 95%, and 100%), cleared in xylene, and embedded in paraffin blocks. Serial sections (4 μm thickness) were cut, mounted on glass slides, and dried overnight at 60 °C. The sections were deparaffinized in xylene and rehydrated in decreasing concentrations of ethanol. Subsequently, the slides were stained with hematoxylin for 5–8 min to visualize nuclei, rinsed in running water, differentiated in 1% acid alcohol, and blued in 0.5% ammonia water or Scott’s tap water. After counterstaining with eosin for 2–3 min to highlight the cytoplasm and extracellular matrix, the slides were dehydrated in graded ethanol, cleared in xylene, and mounted with coverslips for subsequent pathological examination.

### 2.17. Statistical Analysis

All experiments were repeated at least three times. Data are presented as mean ± SD. Student’s *t*-test and analysis of variance (ANOVA) were performed using GraphPad Prism 9.5. Survival analysis was performed using the Kaplan–Meier method. *p* < 0.05 was considered statistically significant (*p* < 0.05; *p* < 0.01; *** *p* < 0.001; **** *p* < 0.0001).

## 3. Results

### 3.1. PZ2 Exerts Potent and Selective Anti-Leukemic Activity In Vitro

**Figure 1:**
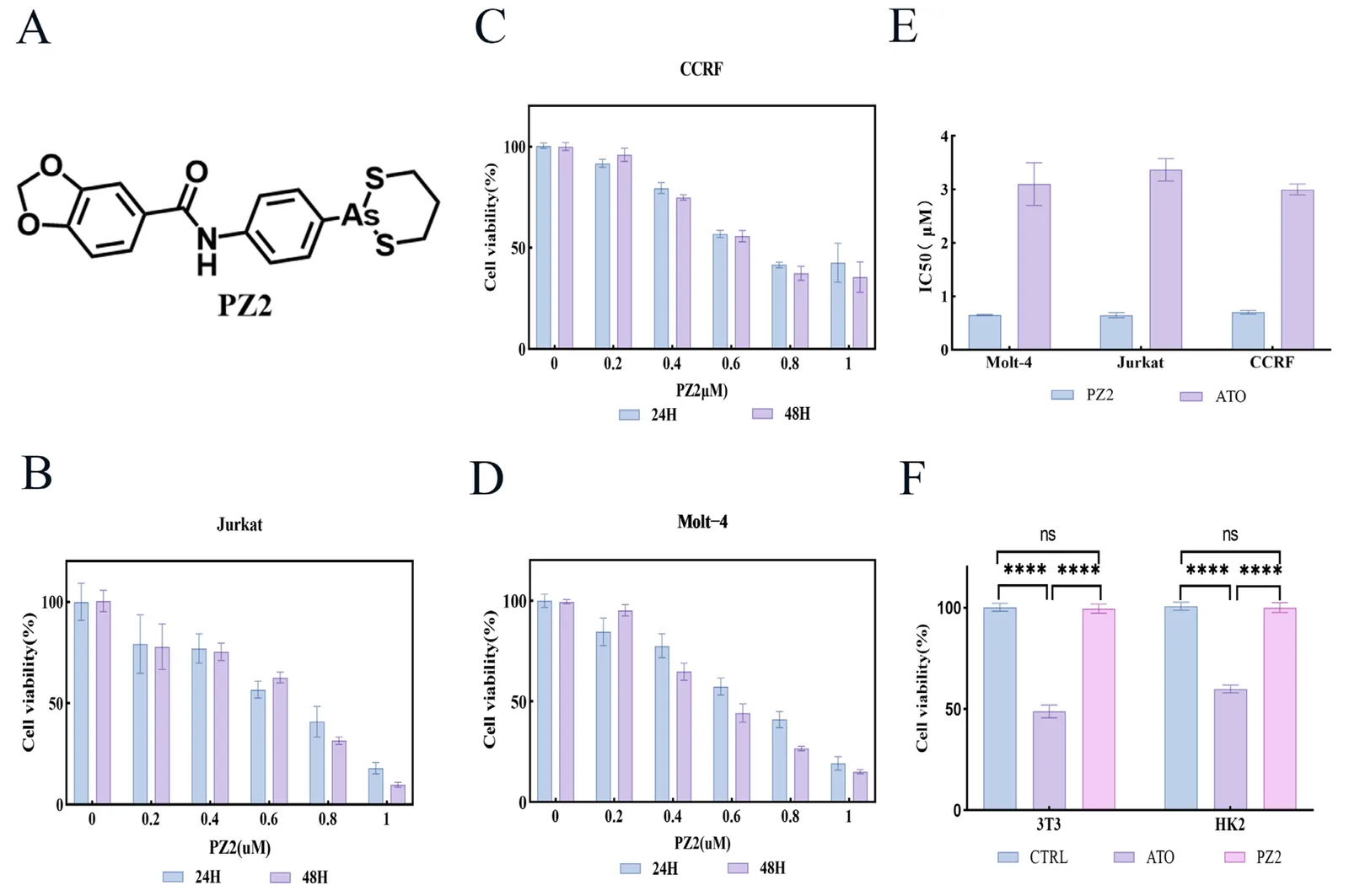
PZ2 exhibits potent and selective antiproliferative activity against T-ALL cells. (**A**) Chemical structure of the novel organoarsenical compound PZ2. (**B**–**D**) Cell viability of Jurkat, Molt-4, and CCRF-CEM cells treated with varying concentrations of PZ2 for 24–48 h, assessed via CCK-8 assay. PZ2 demonstrates time- and dose-dependent cytotoxicity. (**E**) Comparison of half-maximal inhibitory concentration (IC_50_) values of the organic arsenical PZ2 versus the inorganic arsenic drug ATO in T-ALL cell lines. (**F**) Cytotoxicity evaluation of PZ2 on non-malignant cells to assess in vitro safety. Data are presented as the mean ± SD of three independent experiments. **** *p* < 0.0001; ns, not significant.

To establish the therapeutic potential of the novel organic arsenical PZ2 (Figure 1A), we first evaluated its anti-proliferative capacity against T-ALL cell lines. PZ2 exerted significant cytotoxicity in a time- and dose-dependent manner across Jurkat, Molt-4, and CCRF cells (Figure 1B–D). The calculated 24-h IC_50_ values were 0.63 μM, 0.61 μM, and 0.64 μM, respectively. Notably, PZ2 demonstrated significantly higher potency than the clinical-grade inorganic arsenical (ATO), which required a substantially higher concentration (IC_50_ ≈ 3 μM) to achieve comparable inhibition (Figure 1E). To assess the therapeutic index and safety profile, we compared the cytotoxicity of 0.6 μM PZ2 versus ATO in normal cell lines (HK2 and 3T3). While ATO induced profound cellular damage in normal cells, PZ2 exhibited negligible toxicity, yielding a remarkably high selectivity index (defined as IC50 _normal cells_/IC50_T-ALL cells_) of 9.5 on normal cell lines that significantly outperformed ATO and underscored a highly favorable safety window for this novel compound (Figure 1F).

### 3.2. Transcriptomic Profiling Reveals PZ2-Induced Cell Cycle Arrest and Apoptosis

**Figure 2:**
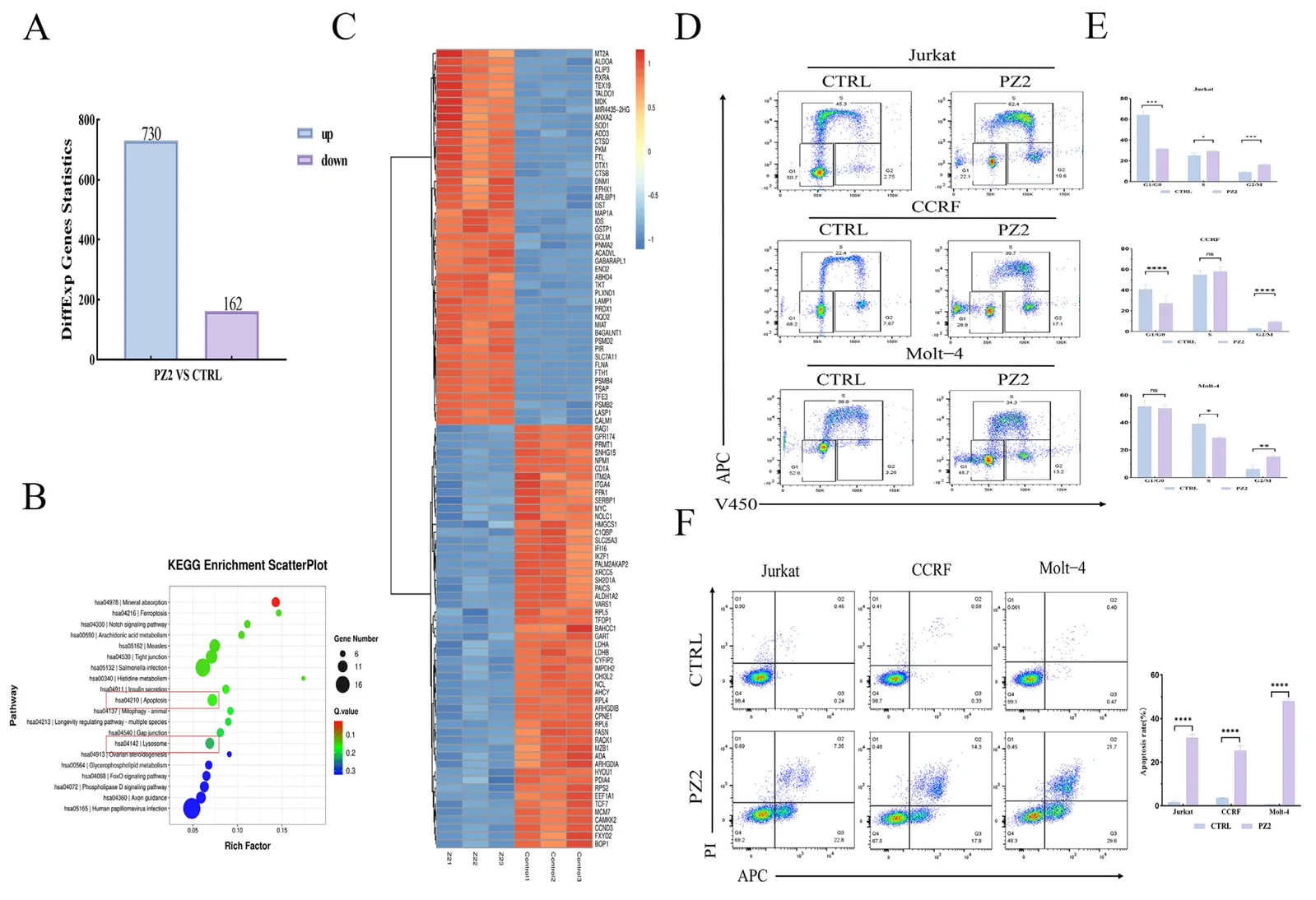
Transcriptomic profiling reveals PZ2-induced cell cycle arrest and apoptosis in T-ALL cells. (**A**) Bar graph quantifying the number of differentially expressed genes (DEGs) following PZ2 treatment. (**B**) Bubble plot of KEGG pathway enrichment analysis for DEGs. The *x*-axis represents the-−log_10_(*p*-value), with bubble size indicating the number of enriched genes per pathway (input number). Bubble color transitions from blue to red to reflect an increasing enrichment ratio (rich factor). (**C**) Hierarchical clustering heatmap of DEGs, with relative expression levels scaled from blue (low expression) to red (high expression). (**D**,**E**) Cell cycle distribution analysis via flow cytometry, demonstrating significant G2/M phase arrest in Jurkat, Molt-4, and CCRF-CEM cells post-treatment. (**F**) Flow cytometric quantification of cellular apoptosis using Annexin V/PI staining, showing an increased proportion of early and late apoptotic cells following PZ2 exposure. Data are presented as the mean ± SD (*n* = 3). * *p* < 0.05, ** *p* < 0.01, *** *p* < 0.001, **** *p* < 0.0001; ns, not significant.

To comprehensively elucidate the molecular mechanisms underlying PZ2-mediated cytotoxicity, we performed transcriptomic profiling in Molt-4 cells. RNA-seq analysis identified 893 differentially expressed genes (730 upregulated, 163 downregulated) following PZ2 treatment (Figure 2A and Figures S1–S3). KEGG pathway enrichment analysis highlighted robust alterations in the apoptosis and autophagy–lysosome signaling pathways (Figure 2B), with core regulatory genes clustered in the expression heatmap (Figure 2C). Guided by these transcriptomic insights, we functionally validated the cell cycle and apoptotic phenotypes. EdU-based cell cycle analysis demonstrated that PZ2 effectively induced marked G2/M phase arrest in T-ALL cells (Figure 2D,E). Concurrently, Annexin V-APC/PI double staining confirmed a significant, dose-dependent induction of apoptosis following PZ2 exposure (Figure 2F).

### 3.3. PZ2 Triggers ROS-Dependent Intrinsic Mitochondrial Apoptosis

**Figure 3:**
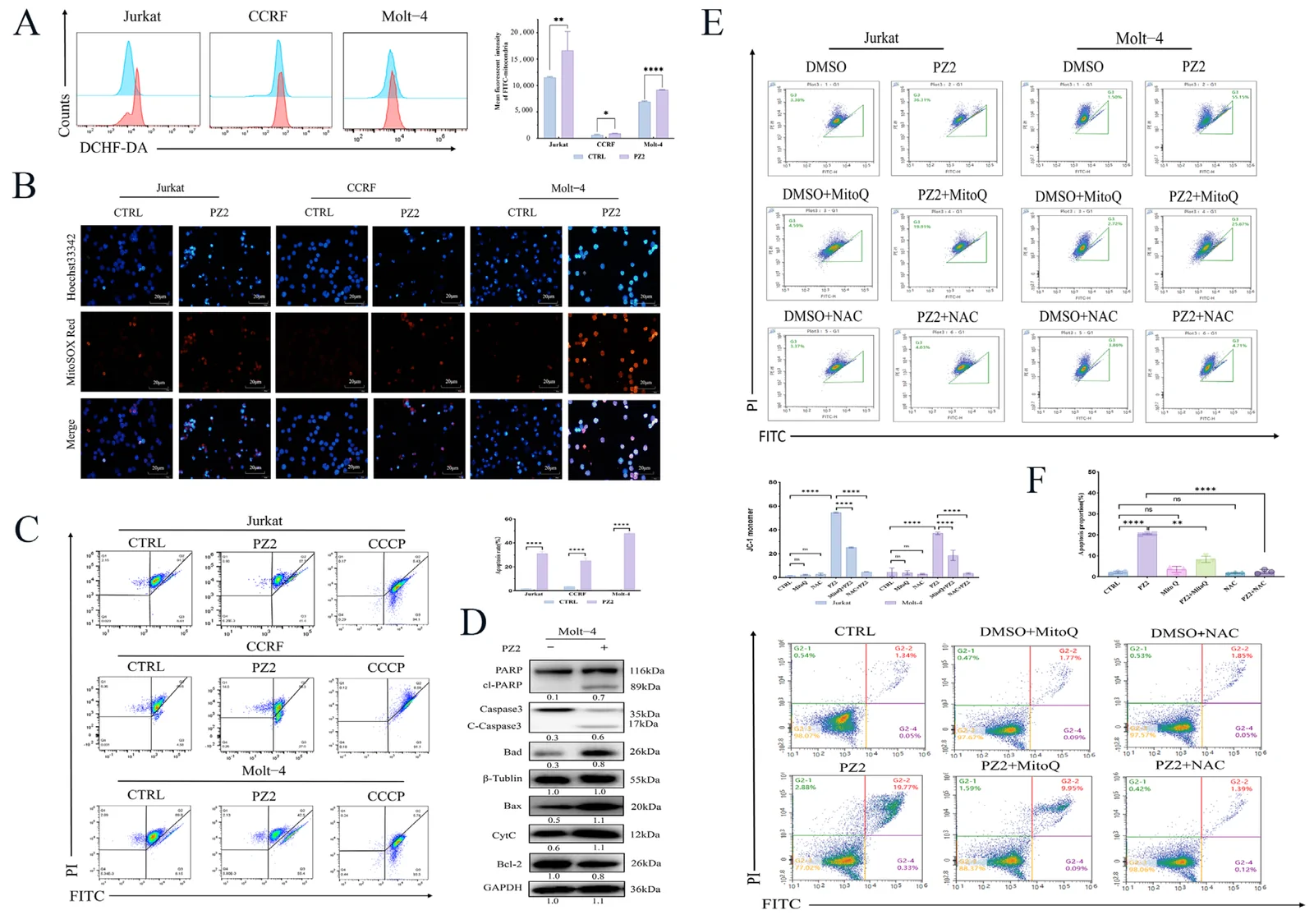
PZ2 triggers ROS-dependent intrinsic mitochondrial apoptosis. (**A**,**B**) Flow cytometric quantification (**A**) and immunofluorescence imaging (**B**) of mitochondrial reactive oxygen species (ROS) levels in T-ALL cells treated with PZ2 (0.6 μM). (**C**) Assessment of mitochondrial membrane potential (ΔΨm) via JC-1 staining. The accumulation of JC-1 monomers indicates severe ΔΨm depolarization. (**D**) Western blot analysis revealing the upregulation of pro-apoptotic proteins (BAD, BAX), downregulation of the anti-apoptotic protein (BCL-2), and increased expression of caspase-dependent cascade markers (cytochrome c, cleaved caspase-3, and cleaved PARP). (**E**) Restoration of ΔΨm following co-treatment with PZ2 and ROS scavengers, evidenced by a reduced JC-1 monomer population. (**F**) Flow cytometric analysis showing that ROS scavenging significantly reverses PZ2-induced apoptosis. Data are presented as the mean ± SD (*n* = 3). * *p* < 0.05, ** *p* < 0.01, **** *p* < 0.0001; ns, not significant. Original western blots are presented in File S1.

Given the established link between arsenicals and oxidative stress, we investigated whether PZ2-induced apoptosis was driven by a redox imbalance. Flow cytometry and fluorescence microscopy revealed a massive intracellular accumulation of reactive oxygen species (ROS) in T-ALL cells treated with 0.6 μM PZ2 for 24 h (Figure 3A,B). This oxidative burst was accompanied by a catastrophic collapse of the MMP, as indicated by the increased JC-1 monomer ratio (Figure 3C). Western blot analysis confirmed the activation of the intrinsic apoptotic cascade, evidenced by the upregulation of pro-apoptotic markers (BAX and BAD), downregulation of anti-apoptotic BCL-2, and the subsequent cleavage of caspase-3 and PARP (Figure 3D). Crucially, co-treatment with the broad-spectrum antioxidant NAC or the mitochondria-targeted ROS scavenger MitoQ significantly reversed MMP depolarization (Figure 3E) and substantially attenuated the apoptotic rate (Figure 3F). These data confirm that PZ2 fundamentally relies on excessive mitochondrial oxidative stress to execute caspase-dependent apoptosis.

### 3.4. PZ2 Promotes Autophagic Cell Death via GABARAPL1 Up-Regulation

**Figure 4:**
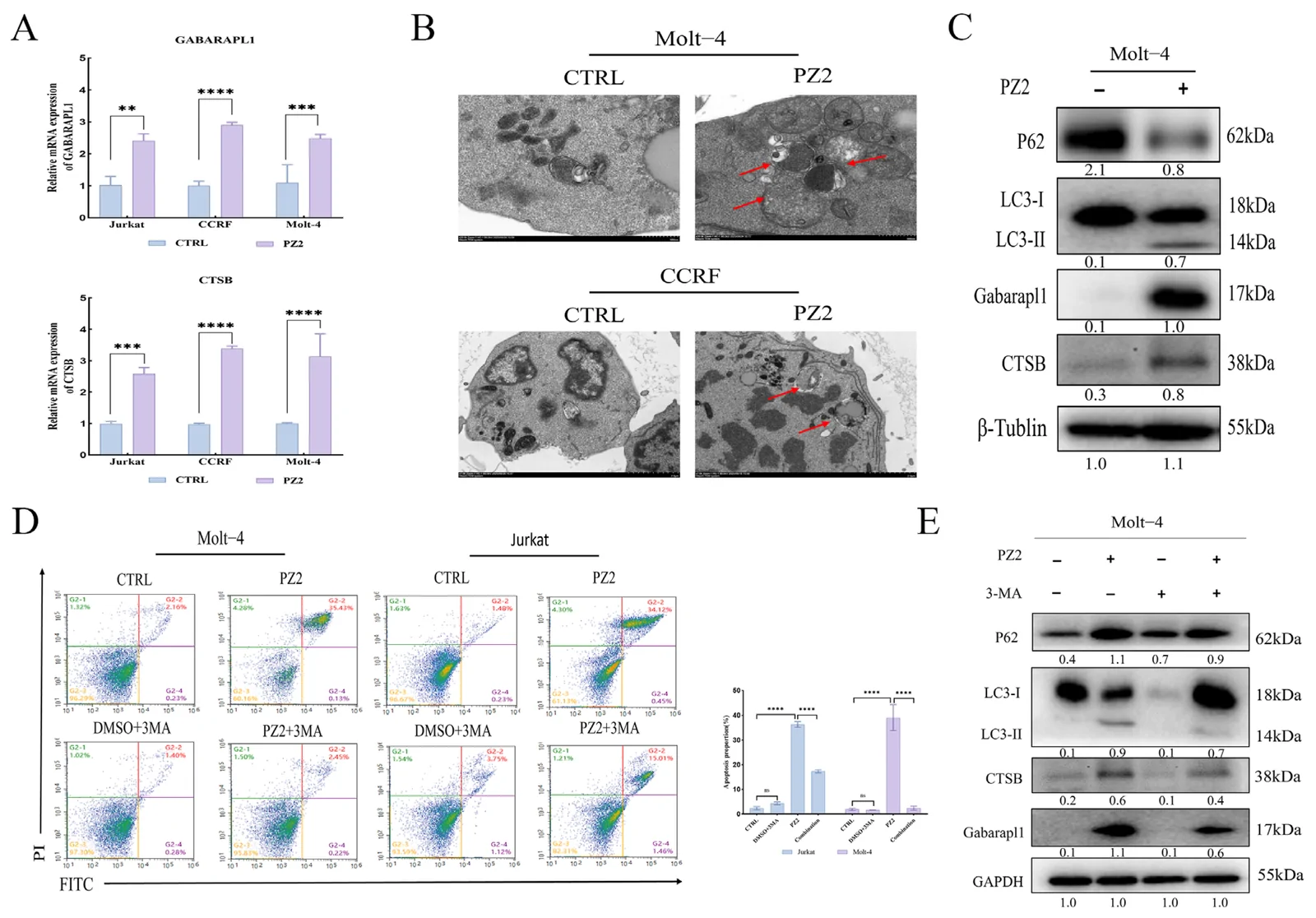
PZ2 induces pro-apoptotic autophagy via the upregulation of GABARAPL1. (**A**) qRT−PCR analysis of the autophagy-related genes *GABARAPL1* and *CTSB* following PZ2 treatment. (**B**) Transmission electron microscopy (TEM) images revealing the pronounced accumulation of autophagic vesicles in PZ2-treated cells. (**C**) Western blot analysis of key autophagy markers, demonstrating increased expression of GABARAPL1, CTSB, and LC3-II, alongside p62 degradation. (**D**) Apoptosis rate assessment following co-treatment with PZ2 and the autophagy inhibitor 3-MA. The combination significantly reduced the apoptosis rate compared to PZ2 monotherapy. (**E**) Western blot analysis confirming the suppression of PZ2-induced autophagic protein expression by 3-MA. Data are presented as the mean ± SD (*n* = 3). ** *p* < 0.01, *** *p* < 0.001, **** *p* < 0.0001; ns, not significant. Original western blots are presented in File S1.

Parallel to apoptosis, our transcriptomic data heavily implicated the autophagy–lysosome axis. Quantitative PCR confirmed that the mRNA levels of autophagy-related genes *GABARAPL1* and *CTSB* were significantly elevated following PZ2 treatment (Figure 4A). Transmission electron microscopy (TEM) corroborated this by revealing a marked increase in the density of double-membrane autophagic vesicles (Figure 4B). Immunoblotting further demonstrated enhanced autophagic flux, characterized by elevated expressions of GABARAPL1, CTSB, and LC3-II, alongside a concomitant degradation of p62 (Figure 4C). To determine whether this robust autophagic response was cytoprotective or pro-death, we utilized the pharmacological autophagy inhibitor 3-MA. Notably, blockade of autophagosome formation with 3-MA significantly rescued T-ALL cells from PZ2-induced apoptosis (Figure 4D) and reversed the expression of *GABARAPL1* and related autophagic markers (Figure 4E). These results suggest that PZ2 induces pro-death autophagy, in which GABARAPL1 may be involved.

### 3.5. PZ2 Robustly Suppresses T-ALL Progression In Vivo

**Figure 5:**
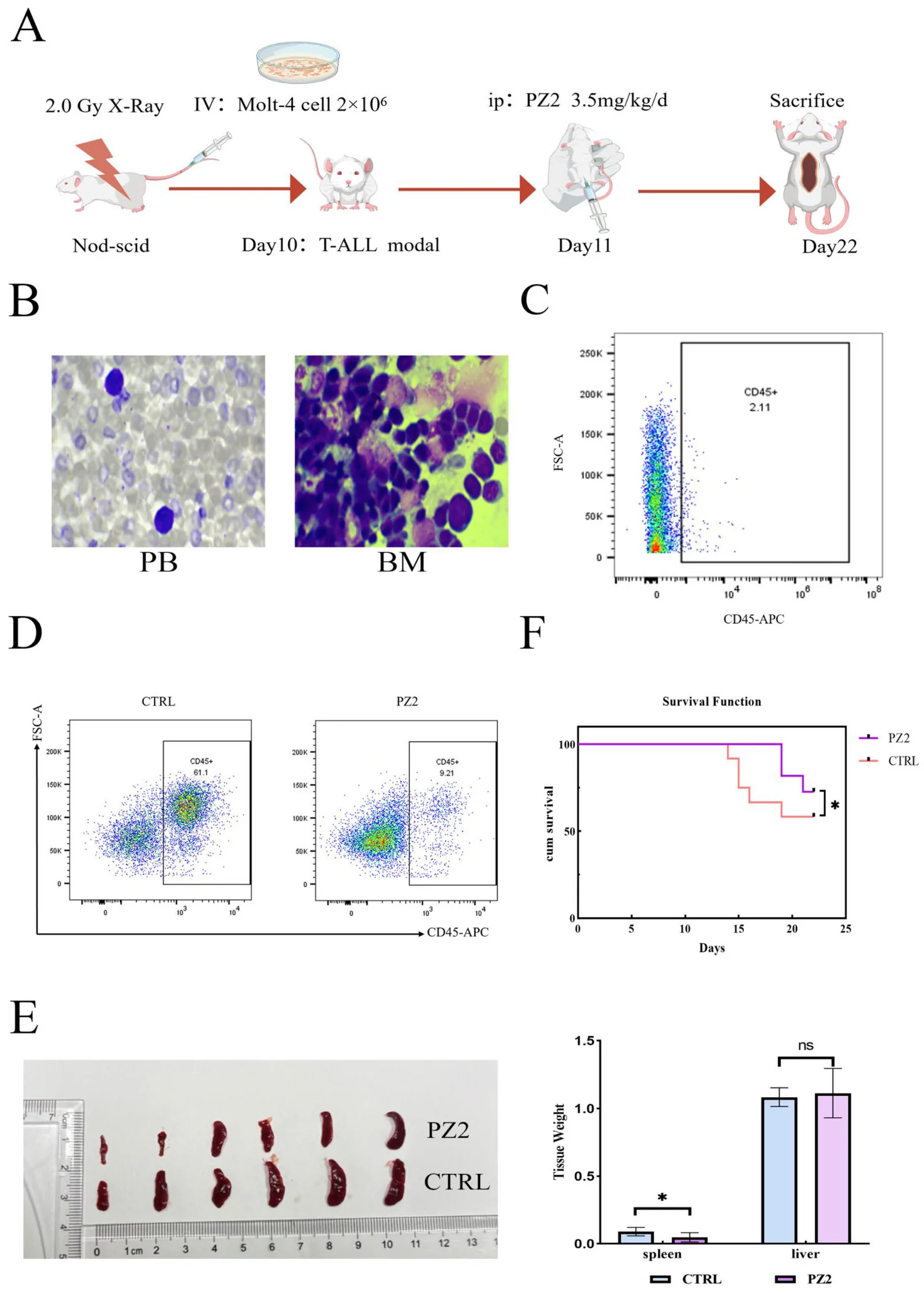
PZ2 exhibits robust anti-leukemic efficacy in a T-ALL cell-derived xenograft (CDX) model. (**A**) Schematic timeline of CDX mouse model establishment and the PZ2 treatment regimen. (**B**) Representative morphological observation of leukemic blasts in murine bone marrow (BM) and peripheral blood (PB) smears on day 10 post-engraftment (100× magnification). (**C**,**D**) Flow cytometric detection (**C**) and quantification (**D**) of human CD45^+^ cell engraftment, demonstrating a significant reduction in bone marrow tumor burden in the PZ2-treated group compared to the vehicle control. (**E**) Gross morphology of spleens from different treatment groups. PZ2 administration significantly ameliorated disease-associated splenomegaly compared with the vehicle-treated control group. (**F**) Kaplan–Meier overall survival curves of the vehicle and PZ2-treated mouse cohorts. * *p* < 0.05, ns, not significant.

To translate our in vitro findings, we evaluated the therapeutic efficacy of PZ2 using a T-ALL cell-line-derived xenograft (CDX) murine model (Figure 5A). Following sublethal irradiation (2.0 Gy), NOD-SCID mice were intravenously injected with 2 × 10^6^ Molt-4 cells. Successful engraftment was systematically verified prior to treatment via morphological evaluation of peripheral blood and bone marrow smears (Figure 5B). Specifically, flow cytometric analysis revealed a robust enrichment of CD45^+^ cells within the bone marrow, further confirming the successful establishment of the systemic leukemic model (Figure 5C). Upon treatment initiation, mice administered PZ2 (3.5 mg/kg/day) exhibited a dramatic reduction in the leukemia burden within the bone marrow compartment compared to vehicle-treated controls (Figure 5D). Furthermore, macroscopic and quantitative evaluations of the spleen demonstrated that PZ2 effectively mitigated T-ALL-induced splenomegaly without affecting body weight (Figure 5E and Figure S4). Most importantly, PZ2 administration translated into a remarkable survival advantage, substantially prolonging the lifespan of the leukemic mice (Figure 5F).

### 3.6. In Vivo Mechanistic Validation Confirms Systemic Safety and Efficacy

**Figure 6:**
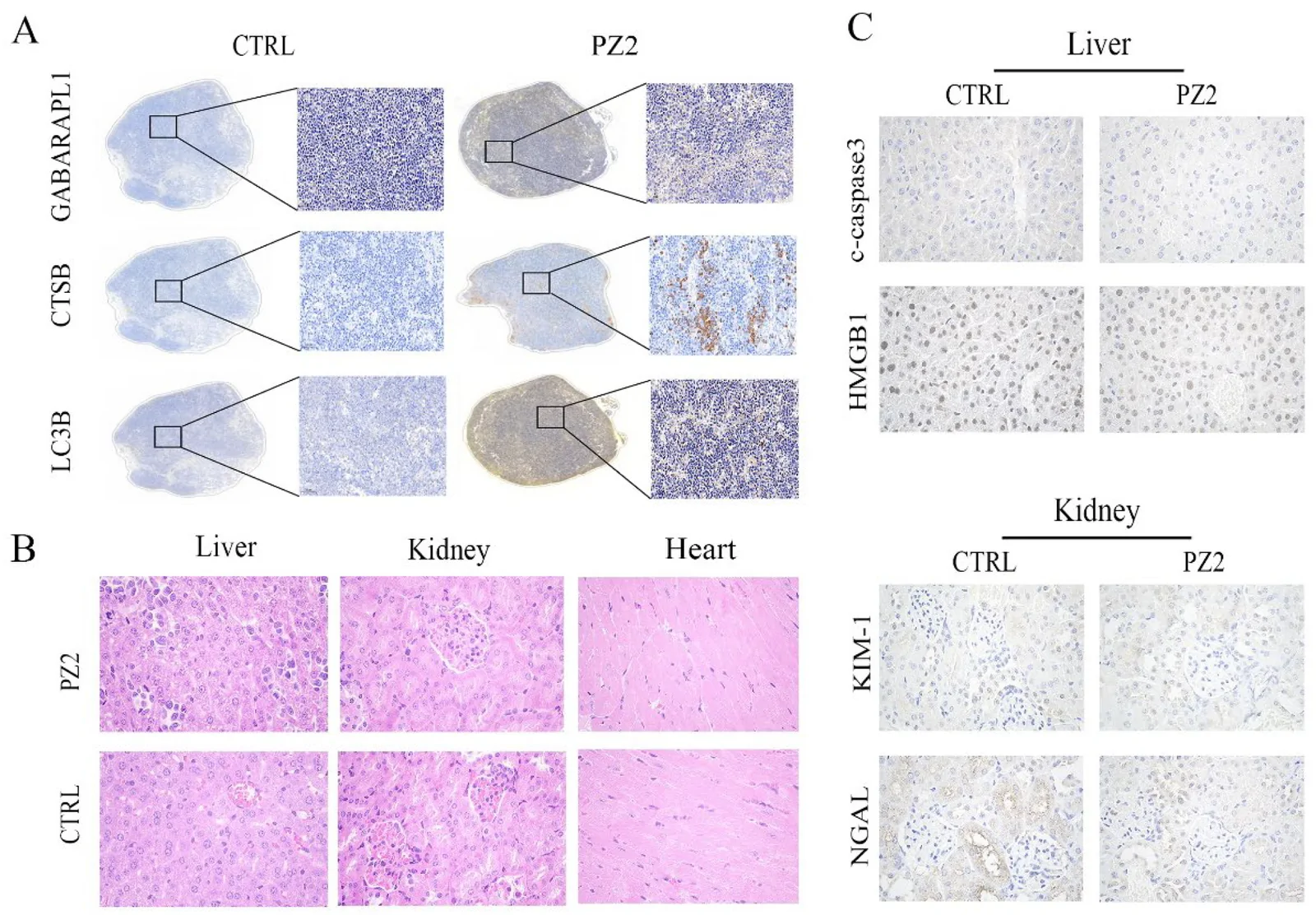
In vivo histopathological evaluation confirms systemic biosafety and target engagement. (**A**) Immunohistochemical (IHC) staining of murine spleen sections, revealing upregulated expression of autophagy-related markers (LC3B, GABARAPL1, and CTSB) following PZ2 treatment. (**B**) Representative hematoxylin and eosin (H&E)-stained sections of the liver, kidney, and heart, demonstrating the absence of discernible histopathological structural differences between the two groups. (**C**) IHC expression analysis of liver injury markers (HMGB1, cleaved caspase-3) and kidney injury markers (NGAL, KIM-1), confirming no significant systemic tissue damage. Data are presented as the mean ± SD (*n* = 3).

Finally, we sought to validate the proposed molecular mechanisms and evaluate the systemic safety of PZ2 in vivo. Immunohistochemical analysis of splenic tissues from the CDX mice perfectly mirrored our in vitro data, showing profound upregulation of GABARAPL1, CTSB, and LC3B within the tumor microenvironment (Figure 6A and Figure S5). To address potential off-target toxicity, we conducted a rigorous histopathological and biochemical evaluation of major metabolic organs. H&E staining revealed no obvious morphological damage or necrotic lesions in the liver and kidneys of PZ2-treated mice (Figure 6B). Consistent with this, the expression levels of critical markers for hepatic injury (HMGB1, cleaved caspase-3) and renal injury (NGAL, KIM-1) remained at baseline, showing no significant differences from the control group (Figure 6C). Collectively, these findings confirm that PZ2 achieves potent anti-leukemic efficacy with a highly favorable in vivo safety profile.

## 4. Discussion

Arsenic, the principal pharmacological component of traditional remedies, has been utilized for centuries under the paradigm of “combating toxicity with toxicity” [23]. Modern oncology has witnessed its landmark translational success, with inorganic arsenic trioxide (ATO) established as a frontline therapy for APL, achieving remarkable complete remission rates of 90–95% [24]. However, its broader clinical application in other hematological malignancies, including T-ALL, is severely constrained by its narrow therapeutic window and systemic toxicity. In this study, PZ2 treatment significantly elevated mitochondrial ROS levels, decreased MMP, increased cytochrome c release, and sequentially activated cleaved caspase-3 and cleaved PARP in T-ALL cells. The use of ROS scavengers partially reversed PZ2-induced apoptosis, directly demonstrating that ROS play a necessary and initiating role in the PZ2-mediated mitochondrial apoptotic pathway.

Mitochondrial dysfunction and oxidative stress are central executioners of arsenical-induced cell death [25]. The classical mode of action for ATO involves binding to vicinal thiol groups of mitochondrial proteins, which provokes a massive accumulation of ROS, prolonged opening of the mitochondrial permeability transition pore, and subsequent activation of the intrinsic apoptotic cascade [26]. Our findings indicate that PZ2 functionally mimics this core pharmacological axis. Following PZ2 treatment, T-ALL cells exhibited a profound oxidative burst, catastrophic loss of mitochondrial membrane potential (MMP), and cytochrome c release, sequentially culminating in caspase-3 and PARP cleavage. Crucially, the application of ROS scavengers (NAC and MitoQ) significantly attenuated PZ2-induced apoptosis, confirming that mitochondrial ROS act as the indispensable initiating signal for PZ2-mediated cytotoxicity.

While basal autophagy typically functions as a cytoprotective mechanism enabling cancer cells to cope with therapeutic stress, hyperactivated autophagy can directly execute autophagic cell death [27]. Beyond classical apoptosis, the most striking finding of this study is the robust induction of pro-death autophagy by PZ2. In contrast to ATO, which induces autophagy via an mTOR-dependent pathway to trigger ETosis, a distinct cell death program that eliminates leukemia-initiating cells in APL [28], our findings unveil a novel, alternative autophagy-activating signaling axis for organic arsenicals. Following PZ2 exposure, we observed a massive accumulation of autophagic vesicles and an enhanced, intact autophagic flux, fundamentally driven by the marked transcriptional and translational upregulation of *GABARAPL1*. Deviating from the classic “pro-survival autophagy” paradigm, pharmacological blockade of autophagosome formation effectively rescued T-ALL cells from PZ2-induced apoptosis. This compellingly demonstrates that *GABARAPL1*-mediated autophagy acts synergistically with, rather than antagonistically against, the apoptotic cascade. Importantly, our findings regarding ROS-driven mitochondrial apoptosis and synergistic stress-induced autophagy align conceptually with recent advances highlighting the therapeutic potential of exploiting cellular stress networks in leukemia. For instance, a recent elegant study demonstrated that targeting mTORC1 engages the PERK-ATF4-BMF integrated stress response, effectively sensitizing subsets of T-ALL cells to venetoclax by priming the mitochondrial apoptotic machinery [29]. While that study focuses on endoplasmic reticulum/unfolded protein stress, our work delineates a parallel paradigm where PZ2 induces overwhelming redox stress. Although initiated through distinct upstream sensors, both strategies ultimately converge on the catastrophic collapse of mitochondrial integrity and the activation of interconnected death programs. Such a “dual activation” strategy offers a profound therapeutic advantage over traditional agents that target a single cell death pathway, providing a highly effective mechanism to bypass the apoptotic resistance frequently encountered in relapsed/refractory T-ALL.

**Figure 7:**
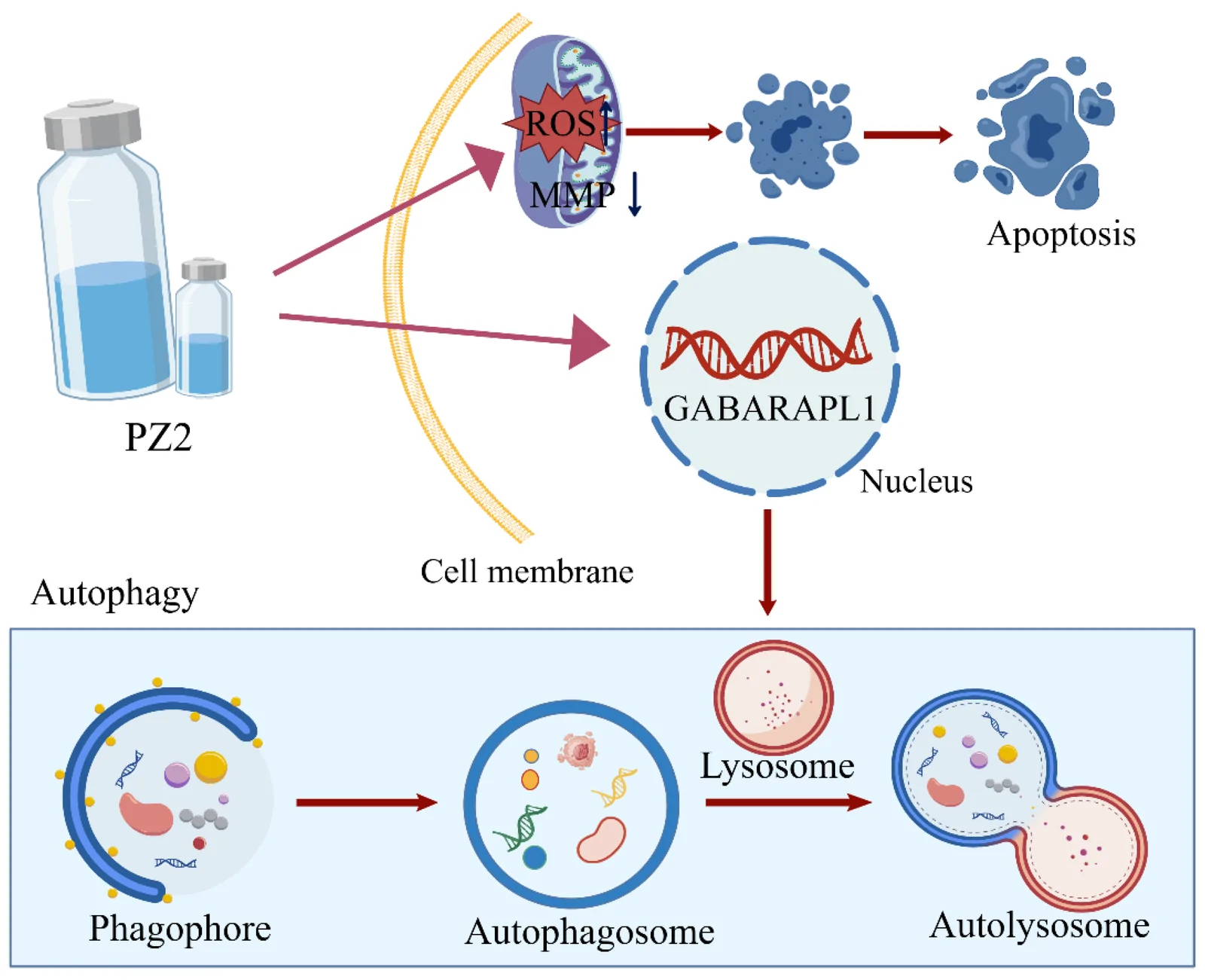
Schematic illustration of the dual signaling pathways driving PZ2-mediated T-ALL cell death. PZ2 synergistically eradicates T-ALL blasts by orchestrating ROS-dependent intrinsic mitochondrial apoptosis and *GABARAPL1*-driven pro-death autophagy.

Although the inorganic arsenical ATO has demonstrated broad-spectrum inhibitory effects against various solid tumors and hematological malignancies [18], its widespread clinical application remains severely hindered by significant systemic toxicity. Consequently, organic arsenicals have emerged as a promising new generation of arsenic-based anti-tumor agents. Unlike their inorganic counterparts, which exhibit relatively indiscriminate targeting, organic arsenicals benefit from rational molecular design and structural modifications. This allows for the precise modulation of specific cellular targets or signaling pathways, thereby preserving potent anti-tumor efficacy while substantially mitigating off-target toxicity in normal cells [30,31]. To date, several organic arsenicals have exhibited remarkable therapeutic activity, particularly in hematological malignancies: A-Z2 induces mitochondrial ROS generation to trigger intrinsic apoptosis in acute myeloid leukemia (AML) [32]; MZ2 reprograms glucose metabolism by inhibiting PDK2 in AML [21] and CZ2 exerts anti-tumor effects in diffuse large B-cell lymphoma by restoring *TMS1* expression via DNA demethylation [33]. Similarly, in solid tumors, AAZ2 targets PDK1 to promote ROS accumulation and mitochondria-dependent apoptosis in gastric cancer cells [20], while MER1 demonstrates superior potency compared to ATO in triggering ROS-driven cell death [34]. Collectively, these studies delineate a unifying mechanistic paradigm for organic arsenicals: ROS-driven mitochondrial intrinsic apoptosis. Our current study firmly positions PZ2 as a highly potent addition to this pharmacological class. However, PZ2 uniquely expands this established paradigm by simultaneously hyperactivating the *GABARAPL1*-driven autophagic axis. This dual action establishes a highly efficient apoptosis–autophagy crosstalk network that proves exceptionally lethal to T-ALL cells (Figure 7).

Despite these promising findings, several limitations of this study should be acknowledged, which also point to important directions for future research. First, most of the mechanistic experiments, including ROS detection, MMP analysis, autophagy marker evaluation, and caspase activation assays, were performed primarily in MOLT-4 cells. Although the other two T-ALL cell lines, Jurkat and CCRF-CEM, were used to confirm the cytotoxic effects of PZ2, the full mechanistic validation in these cell lines was not completed due to time and resource constraints. Second, primary T-ALL samples or patient-derived xenograft models were not included in this study because of restricted access to patient samples. Therefore, the translational claims should be interpreted with caution, and future investigations using additional T-ALL cell lines and patient-derived models are necessary to validate the mechanistic findings and to further assess the clinical potential of PZ2. Third, the direct molecular target of PZ2 remains to be elucidated. Likewise, the upstream epigenetic or transcriptional regulators driving *GABARAPL1* overexpression, as well as the complex crosstalk between mitochondrial ROS and autophagic flux, require further dissection. Future studies employing chemoproteomics and CRISPR/Cas9-mediated knockout models will be essential to map the complete molecular interactome of PZ2. Fourth, the selectivity of PZ2 was evaluated only in HK2 and 3T3 cells, which do not represent normal hematopoietic populations; its toxicity in primary normal T cells and CD34+ HSPCs therefore remains to be determined. Given that these cell populations are critical for assessing the therapeutic window, future studies using primary human peripheral blood T cells and CD34+ HSPCs are needed to define the selectivity and therapeutic index of PZ2. Finally, evaluating the in vivo synergistic potential of PZ2 in combination with existing chemotherapeutic or targeted agents would help accelerate its clinical translation.

## 5. Conclusions

In conclusion, this study systematically characterizes PZ2, a novel organic arsenical, as a highly potent and safe therapeutic candidate for T-ALL. PZ2 circumvents the systemic toxicity associated with traditional inorganic arsenic while achieving superior anti-leukemic efficacy. Mechanistically, PZ2 eliminates T-ALL cells through two synergistic death programs: ROS-driven mitochondrial apoptosis and pro-death autophagy potentially involving *GABARAPL1*. Our findings not only expand the pharmacological spectrum of organic arsenicals but also highlight the therapeutic potential of targeting the apoptosis–autophagy crosstalk network, offering a promising new strategy for overcoming drug resistance in aggressive hematological malignancies.

## Data Availability

Data will be made available on request.

## Supplementary Materials

The following supporting information can be downloaded at: https://www.mdpi.com/article/10.3390/cancers18193150/s1, Figure S1: Sequencing quality information; Figure S2: PCA analysis; Figure S3: GSEA analysis; Figure S4: Body weight curve of mice; Figure S5: Quantification of immunohistochemistry. File S1: Original uncropped Western blot images.
