# Supplementary material for: The Novel Organic Arsenical PZ2 Exhibits Potent Anti-T-ALL Activity via ROS-Dependent Apoptosis and GABARAPL1-Induced Autophagic Cell Death
Source: Cancers (Basel). 2026 Sep 29;18(19):3150. doi: 10.3390/cancers18193150 (PMC13664214; doi:10.3390/cancers18193150)

Figure S1 Sequencing quality information

Sample	Valid reads	Mapped reads	Unique Mapped reads	Multi Mapped re	PE Mapped r	Reads m	Reads map to antiser	Non-splice re	Splice reads
Control	33889758	32078268(94.65%)	31290863(92.33%)	787405(2.32%)	29297910(86	1561217	15678689(46.26%)	17851109(52.6	13439754(39.66%)
Control	38783462	37003608(95.41%)	36076512(93.02%)	927096(2.39%)	34577332(89	1800931	18067202(46.58%)	20518362(52.9	15558150(40.12%)
Control	38779938	36903135(95.16%)	35973127(92.76%)	930008(2.40%)	33836922(87	1795543	18017692(46.46%)	20436626(52.7	15536501(40.06%)
Jcontro	40138330	38224357(95.23%)	37270978(92.86%)	953379(2.38%)	35626422(88	1860262	18668352(46.51%)	21232328(52.9	16038650(39.96%)
Jcontro	41664996	39712343(95.31%)	38725945(92.95%)	986398(2.37%)	36317188(87	1933358	19392356(46.54%)	22235184(53.3	16490761(39.58%)
Jcontro	39853438	37935667(95.19%)	36979349(92.79%)	956318(2.40%)	34620824(86	1845878	18520564(46.47%)	21229043(53.2	15750306(39.52%)
JZ21	34090068	32260080(94.63%)	31458598(92.28%)	801482(2.35%)	29401680(86	1569147	15767125(46.25%)	18453851(54.1	13004747(38.15%)
JZ22	35696764	33761164(94.58%)	32933510(92.26%)	827654(2.32%)	30843720(86	1642798	16505524(46.24%)	19640406(55.0	13293104(37.24%)
JZ23	39003362	37017271(94.91%)	36111461(92.59%)	905810(2.32%)	33919526(86	1801890	18092552(46.39%)	21475687(55.0	14635774(37.52%)
Z21	39366980	37511157(95.29%)	36575191(92.91%)	935966(2.38%)	34347194(87	1825438	18320804(46.54%)	21130373(53.6	15444818(39.23%)
Z22	37727108	35952232(95.30%)	35044163(92.89%)	908069(2.41%)	33093290(87	1749174	17552414(46.52%)	20358107(53.9	14686056(38.93%)
Z23	39913152	38035504(95.30%)	37095374(92.94%)	940130(2.36%)	35086488(87	1851405	18581318(46.55%)	21615247(54.1	15480127(38.78%)
Z2V1	35302146	33668421(95.37%)	32858518(93.08%)	809903(2.29%)	31696106(89	1640540	16453110(46.61%)	19381013(54.9	13477505(38.18%)
Z2V2	40036536	38117021(95.21%)	37159286(92.81%)	957735(2.39%)	35153800(87	1854446	18614820(46.49%)	21573783(53.8	15585503(38.93%)
Z2V3	38301666	36529861(95.37%)	35605917(92.96%)	923944(2.41%)	33589414(87	1777272	17833189(46.56%)	20547112(53.6	15058805(39.32%)

Sample	Raw Data		Valid Data		Valid Ratio(re	Q20%	Q30%	GC content%
	Read	Base	Read	Base				
Control	35152012	5.27G	33889758	5.08G	96.41	99.71	96.14	47.50
Control	39930156	5.99G	38783462	5.82G	97.13	99.79	96.75	47.50
Control	40233006	6.03G	38779938	5.82G	96.39	99.72	96.62	48
Jcontro	41285440	6.19G	40138330	6.02G	97.22	99.78	96.67	48.50
Jcontro	43227730	6.48G	41664996	6.25G	96.38	99.74	96.91	48.50
Jcontro	41363994	6.20G	39853438	5.98G	96.35	99.72	96.84	48.50
JZ21	35393620	5.31G	34090068	5.11G	96.32	99.70	96.22	48
JZ22	37036430	5.56G	35696764	5.35G	96.38	99.68	96.24	47.50
JZ23	40434564	6.07G	39003362	5.85G	96.46	99.71	96.63	47.50
Z21	40942374	6.14G	39366980	5.91G	96.15	99.73	96.75	47.50
Z22	39170664	5.88G	37727108	5.66G	96.31	99.73	96.71	47.50
Z23	41346062	6.20G	39913152	5.99G	96.53	99.74	96.72	47.50
Z2V1	36429058	5.46G	35302146	5.30G	96.91	99.73	96.79	47.50
Z2V2	41554594	6.23G	40036536	6.01G	96.35	99.72	96.60	47.50
Z2V3	39725150	5.96G	38301666	5.75G	96.42	99.75	96.90	47.50

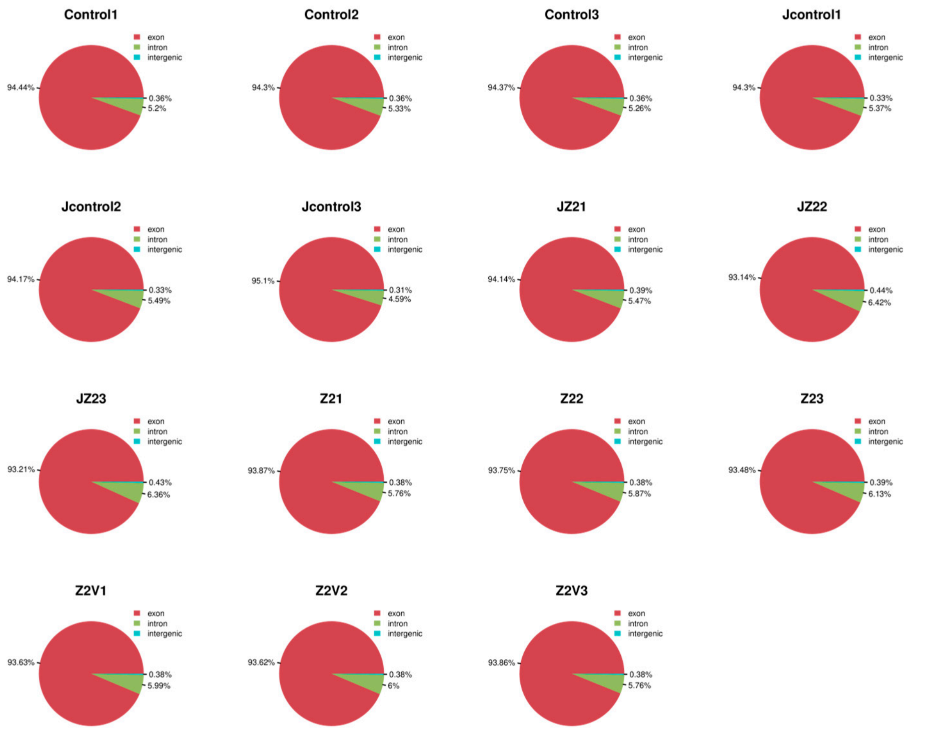

Figure S2: PCA analysis

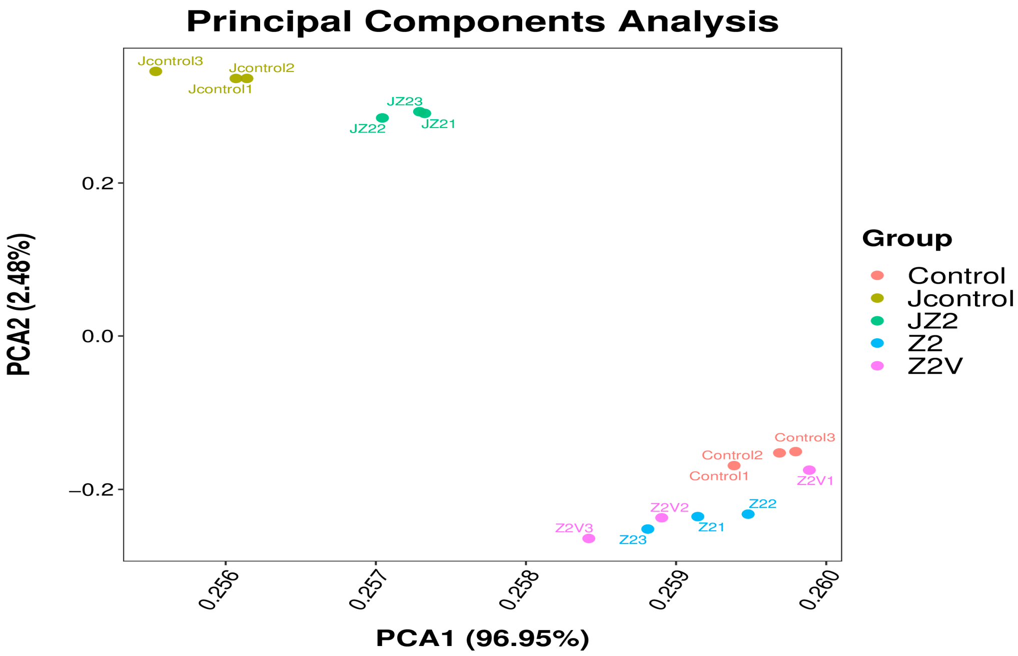

Figure S3: GSEA analysis.

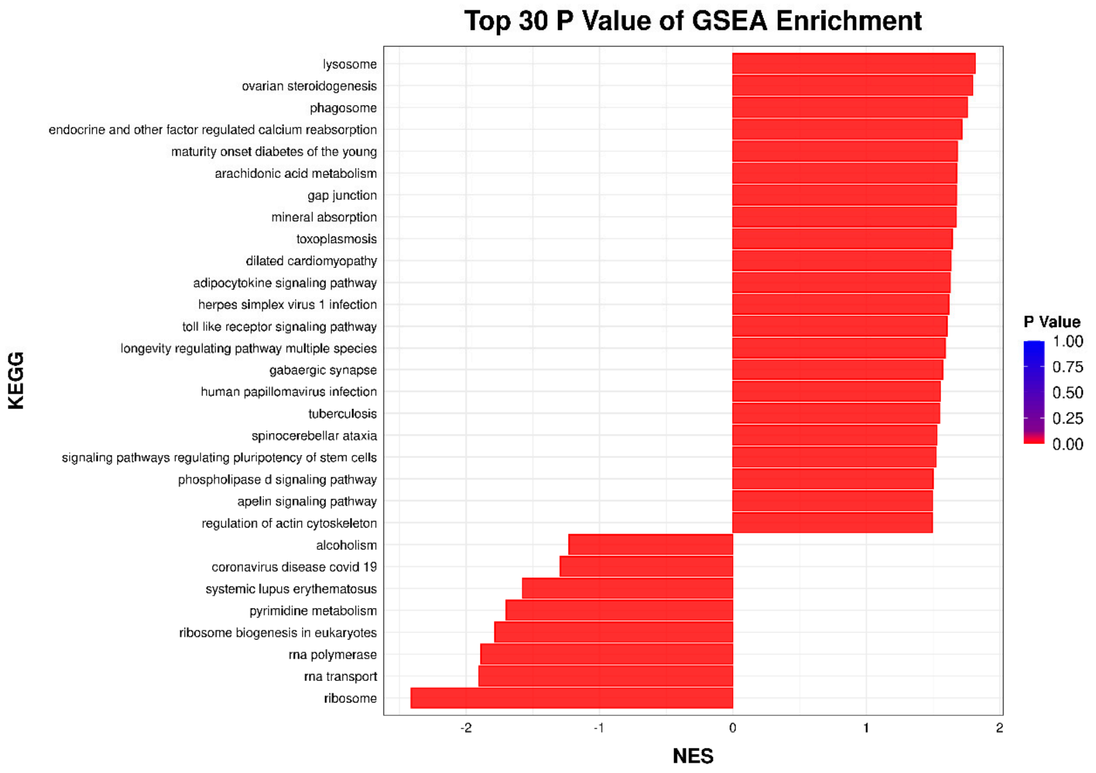

Figure S4: Body weight curve of mice

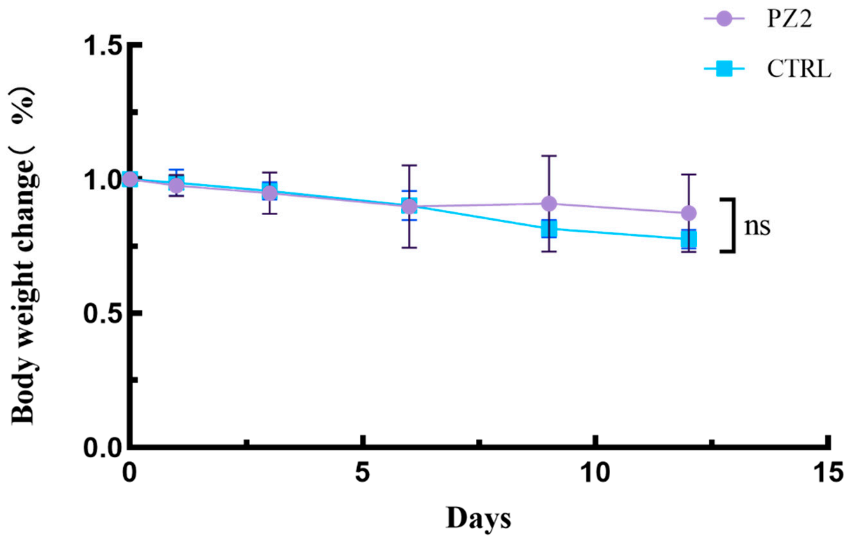

Figure S5: Quantification of immunohistochemistry.

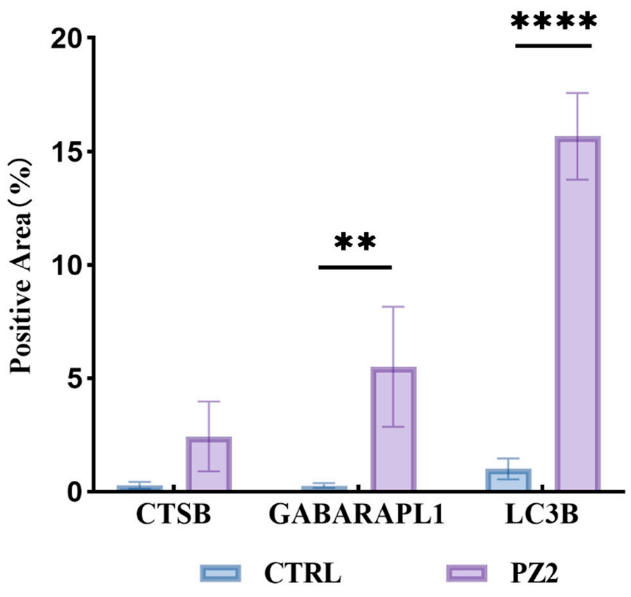

Supplement: Supplementary file 1 [file cancers-18-03150-s001.zip › cancers-4520605-supplementary.pdf]
