# Supplementary material for: The Novel Organic Arsenical PZ2 Exhibits Potent Anti-T-ALL Activity via ROS-Dependent Apoptosis and GABARAPL1-Induced Autophagic Cell Death
Source: Cancers (Basel). 2026 Sep 29;18(19):3150. doi: 10.3390/cancers18193150 (PMC13664214; doi:10.3390/cancers18193150)

c

F3D PARP

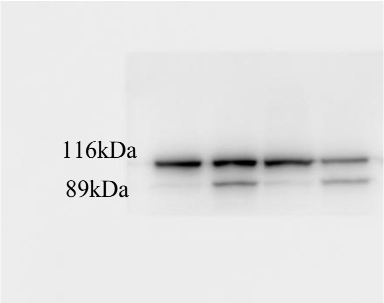

F3D Caspase3

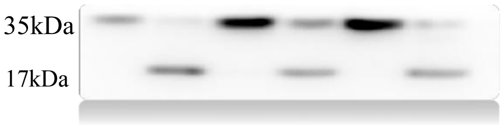

F3D Bad

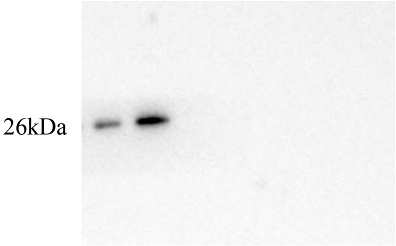

F3D Tublin

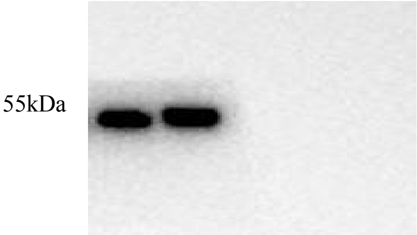

F3D Bax

c

20kDa

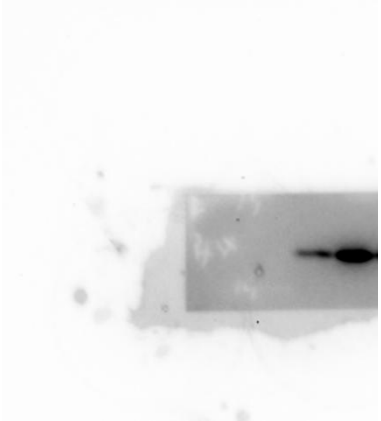

F3D Cyt C

12kDa

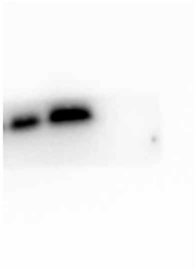

F3D Bcl-2

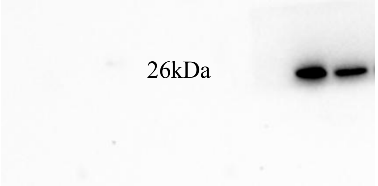

F 3D GAPDH

36kDa

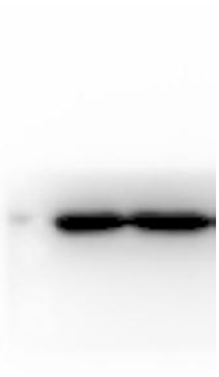

c

F4C P62

62kDa

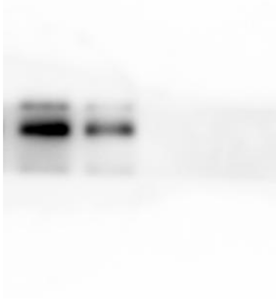

F4C LC3II/I

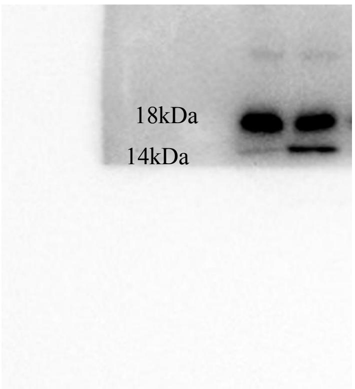

F4C GABARAPL1

17kDa

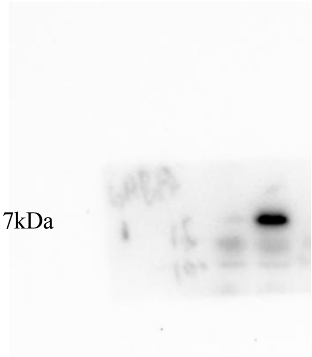

F4C CTSB

38kDa

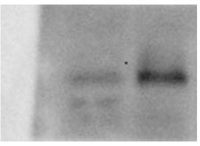

c

F4C Tublin

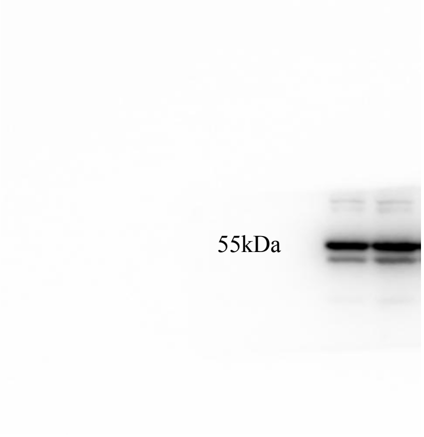

F4E gabarapl1

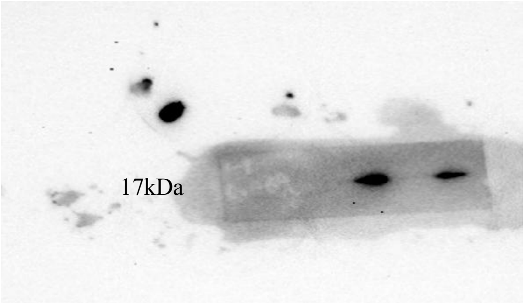

F4E P62

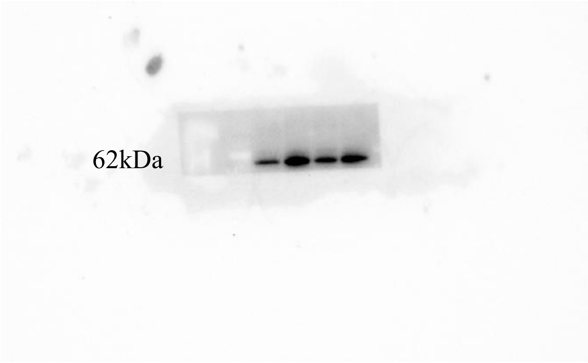

F4E LC3II/I

c

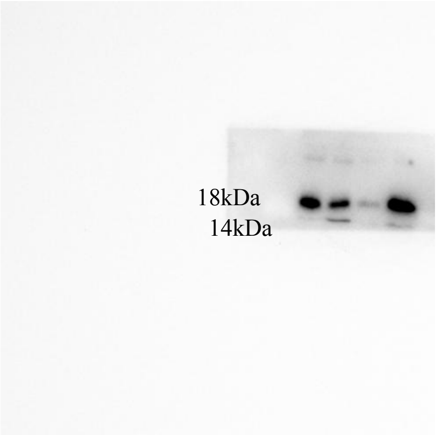

F4C CTSB

38kDa

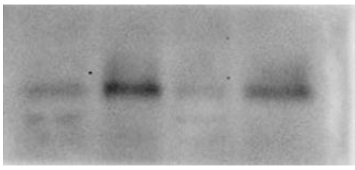

F4E GAPDH

36kDa

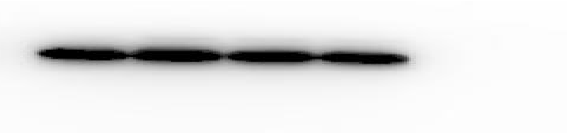

Supplement: Supplementary file 1 [file cancers-18-03150-s001.zip › File S1.pdf]
